# Freedom of Thought and Mental Integrity: The Moral Requirements for Any Neural Prosthesis

**DOI:** 10.3389/fnins.2018.00082

**Published:** 2018-02-19

**Authors:** Andrea Lavazza

**Affiliations:** Neuroethics, Centro Universitario Internazionale, Arezzo, Italy

**Keywords:** neural prosthesis, freedom of thought, mental privacy, thought control, brain datasets

## Abstract

There are many kinds of neural prostheses available or being researched today. In most cases they are intended to cure or improve the condition of patients affected by some cerebral deficiency. In other cases, their goal is to provide new means to maintain or improve an individual's normal performance. In all these circumstances, one of the possible risks is that of violating the privacy of brain contents (which partly coincide with mental contents) or of depriving individuals of full control over their thoughts (mental states), as the latter are at least partly detectable by new prosthetic technologies. Given the (ethical) premise that the absolute privacy and integrity of the most relevant part of one's brain data is (one of) the most valuable and inviolable human right(s), I argue that a (technical) principle should guide the design and regulation of new neural prostheses. The premise is justified by the fact that whatever the coercion, the threat or the violence undergone, the person can generally preserve a “private repository” of thought in which to defend her convictions and identity, her dignity, and autonomy. Without it, the person may end up in a state of complete subjection to other individuals. The following functional principle is that neural prostheses should be technically designed and built so as to prevent such outcomes. They should: (a) incorporate systems that can find and signal the unauthorized detection, alteration, and diffusion of brain data and brain functioning; (b) be able to stop any unauthorized detection, alteration, and diffusion of brain data. This should not only regard individual devices, but act as a general (technical) operating principle shared by all interconnected systems that deal with decoding brain activity and brain functioning.

## Introduction

This is Germany. The Nazi party is in power. Hladík, a Jewish citizen and writer, is arrested and sentenced to death. The thought of being killed terrifies him, but what scares him the most is the idea of not being able to finish what would have been his most important play: *The Enemies*.

“He had asked God for an entire year in which to finish his work: His omnipotence had granted him the time. For his sake, God projected a secret miracle: German lead would kill him, at the determined hour, but in his mind a year would elapse between the command to fire and its execution (…) He disposed of no document but his own memory; the mastering of each hexameter as he added it, had imposed upon him a kind of fortunate discipline not imagined by those amateurs who forget their vague, ephemeral, paragraphs. He did not work for posterity, nor even for God, of whose literary preferences he possessed scant knowledge. (…) He brought his drama to a conclusion: he lacked only a single epithet. He found it: the drop of water slid down his cheek. He began a wild cry, moved his face aside. A quadruple blast brought him down. Jaromir Hladik died on March 29, at 9:02 in the morning” (Borges, *The Secret Miracle*).

Thus wrote Jorge Luis Borges in a famous short story, showing the relevance of our mental life even in the face of the extreme threat—that of the end of life. Borges wrote fiction, introducing references to miracles that are little pertinent here. However, a valid point could still be made: if Nazi criminals, like other evil people in the history of humanity, had come to realize that their victims could find great comfort in thoughts concealed within their minds, they would have tried to stop this by all means. Well, today we might be living in an age where this is possible.

Of course, it is probably fair to say that the research done in universities, corporations, hospitals, and other institutions is geared toward beneficial or neutral purposes, where any abuse is attributable to single malicious individuals or to non-democratic regimes. Nevertheless, the development of the latest devices has increased the possibility that mental/brain privacy and integrity be put at risk in an unprecedented way (cf. Rose, 2005; Rose and Abi-Rached, 2013).

Just to make some examples, we have gone from recording electrical activity by means of EEG in strictly clinical settings to neurofeedback with portable instruments that monitor and modulate the individual's attention to specific tasks (Thibault et al., 2016). We can predict intentions and choices that will be made within a few seconds by means of fMRI (Haynes et al., 2007; Soon et al., 2008, 2013). We can interpret the neural activity of an individual by means of fMRI scans and understand whether he is thinking about, say, a motorcycle or a knife (Kay et al., 2008). We are able to identify forms of awareness thanks to brain activation in subjects that are in an apparent vegetative state (Monti et al., 2010), and we can understand the neural signature of political orientation (Schreiber et al., 2013). Neuromarketing makes it possible to know and/or manipulate someone's taste with various direct or indirect techniques that capture hidden preferences (McClure et al., 2004). In the legal field it seems possible to understand with different methods if subjects are lying (Wolpe et al., 2005; Monaro et al., 2017) and to evaluate the possibility of recidivism by observing the brain profiles of individuals released from prison on parole (Aharoni et al., 2013). There have been fairly successful attempts at obtaining speech from brain waves (Herff et al., 2015; Mirkovic et al., 2015), and we have just opened the door to optogenetics, which has made it possible—in non-human animals—to radically change behaviors or modify memories, implanting new ones (Kim et al., 2013; Redondo et al., 2014; Berndt et al., 2016; Ferenczi et al., 2016). And new micro brain implants for reading or controlling neural activity and new kinds of brain-computer interfaces could be made real by advancements in miniaturizing devices. For example, recently antennas for wireless communication 100 times smaller than their current sizes have been figured out (Nan et al., 2017). In 2017, Darpa launched the Neural Engineering System Design project, aimed to achieve a “wireless human brain device that can monitor brain activity using 1 million electrodes simultaneously and selectively stimulate up to 100,000 neurons” (Yuste et al., 2017).

It therefore seems reasonable to try to avoid potential abuses not only by establishing rights and laws, but also by incorporating in the devices themselves the possibility of preventing these abuses according to a precisely defined technical principle. There are already juridical and deontological tools available, of course, such as the Declaration of Helsinki, the Belmont Report for the United States and the Oviedo Convention for Europe. However, in May 2017 a team of leading scholars in the fields of neurotechnologies (including neural prostheses) and AI, called the Morningside Group, gathered at Columbia University in New York to discuss the ethics of neurotechnologies and machine intelligence. They stated that “the existing ethics guidelines are insufficient for this realm” (Yuste et al., 2017). In their view, “as neurotechnologies develop and corporations, governments and others start striving to endow people with new capabilities, individual identity (our bodily and mental integrity), and agency (our ability to choose our actions) must be protected as basic human rights” (*ib*.). Expressing a certain worry about the use of new enhancement techniques, they urged “that guidelines are established at both international and national levels to set limits on the augmenting neurotechnologies that can be implemented, and to define the contexts in which they can be used—as is happening for gene editing in humans” (*ib*.).

So, first I will argue for the protection of mental/cerebral privacy and integrity; then I will establish a principle that may be valid for all devices that have the ability to detect, spread, and alter (certain) brain data and brain functioning. Some objections may arise about the absolute conception of the right to mental integrity and therefore also to the application of the technical principle of device control. I shall therefore discuss some of these objections.

## Mental/cerebral integrity

A famous text by Henry David Thoreau dated 1849 illustrates the importance of the various aspects of human action that, at the time, fell under the influence of the state.

“I have paid no poll-tax for 6 years. I was put into a jail once on this account, for one night; and, as I stood considering the walls of solid stone, two or three feet thick, the door of wood and iron, a foot thick, and the iron grating which strained the light, I could not help being struck with the foolishness of that institution which treated me as if I were mere flesh and blood and bones, to be locked up. I wondered that it should have concluded at length that this was the best use it could put me to, and had never thought to avail itself of my services in some way. I saw that, if there was a wall of stone between me and my townsmen, there was a still more difficult one to climb or break through before they could get to be as free as I was. I did not for a moment feel confined, and the walls seemed a great waste of stone and mortar. I felt as if I alone of all my townsmen had paid my tax.

They plainly did not know how to treat me, but behaved like persons who are underbred. In every threat and in every compliment there was a blunder; for they thought that my chief desire was to stand the other side of that stone wall. I could not but smile to see how industriously they locked the door on my meditations, which followed them out again without let or hindrance, and they were really all that was dangerous. As they could not reach me, they had resolved to punish my body; just as boys, if they cannot come at some person against whom they have a spite, will abuse his dog. I saw that the State was half-witted, that it was timid as a lone woman with her silver spoons, and that it did not know its friends from its foes, and I lost all my remaining respect for it, and pitied it. Thus the State never intentionally confronts a man's sense, intellectual or moral, but only his body, his senses. It is not armed with superior wit or honesty, but with superior physical strength” (Thoreau, *Resistance to Civil Government)*.

In the situation described by Thoreau, the state's repressive apparatus could not do much against the ideas that supported civil disobedience. But today the scenario is changing. In what has been termed an era of neuroscientific and neurotechnological imperialism and pervasiveness (Frazzetto, and Anker, 2009; Fumagalli, 2018), it seems much easier to violate the privacy and integrity of our brain and minds.

In fact, the consideration and respect for the autonomy and self-determination of the individual have gradually increased in contemporary culture, also in the wake of Kant's deontological reflection and Mill's liberal utilitarianism, making it harder to justify generically paternalistic positions. A clear example can be found in the field of bioethics, where the patient's informed choices have prevalence over medical judgment and community rules. From an epistemological point of view, the premise is that knowledge concerning the facts and dynamics of human relationships is widespread, nobody knows everything or can decide for everyone in the best way.

Privacy is one of the conditions for the exercise of personal freedom and autonomy. The concept of privacy generally regards the protection of a space of non-interference, based on a principle of “inviolate personality” which—as Brandeis and Warren (1890) famously stated—is a part of the person's general right of immunity. Indeed, privacy limits the intrusion into a person's seclusion or solitude or into her private affairs. The right to privacy seems to prevent the public disclosure of embarrassing facts as well as actions that would publicly put one in a bad light. One can argue that, if others can enter your own sphere, even just to see it, this potentially affects your freedom of action, because being “observed” implies giving others an advantage over us, thus creating an asymmetry between us and them, and generally binding and limiting ourselves. The right to privacy thus seems an essential correlate of autonomy, which, as we have noted, is the preferred framework of liberal societies.

Distinguishing, like Berlin (1969), between negative freedom and positive freedom makes it easier to appreciate the value of freedom and the justifications that can be offered for it. Negative freedom has to do with the absence of external obstacles to the agent: we are free if nobody prevents us from doing what we want to do. It is political freedom, in favor of which millions of people have sacrificed themselves throughout the twentieth century. Positive freedom is characterized instead by the control exercised by the agent: one is free if one is self-determined, if one influences one's destiny according to one's own interests. But what is the area in which one should leave the subject to do or be what he is capable of doing or being? What is the source of control or interference that can cause someone to do or be something rather than something else?

According to a radical interpretation of positive freedom, it can be said that in many cases (of divided self, of incompatible desires, of neurological diagnoses of true, or presumed diseases, of activations in the average or above-normal range of a cerebral area), a self can be considered superior to another, or an activation to another, and one can therefore think that one self should take precedence over another. The aim will then be to find the true self of the individual in question. Individuals that are more rational or have more knowledge (or better means) could then act to make the true self and the right neuronal activation prevail in everyone (even simply through a preventive control). Anything could be done in the name of the true self or the right neuronal activation, in the presumed certainty—says Berlin—that everything that represents the ultimate purpose of man (happiness, the fulfillment of duty, wisdom, a just society, self-realization) must be the choice of this “true” self, even if hidden or unexpressed. This is the essence of paternalism at its fullest, which is the antithesis of freedom. And this kind of paternalism has often proven in history to be cruel and ineffective, being unable to increase overall happiness. The freedom and autonomy of the individual, also guaranteed by her mental integrity, are instead the conditions for several other goals in addition to being an objective in itself, as written today in the Constitutions of many democratic countries.

Here it is worth clarifying the different elements that are at stake and the way in which they are involved. Any individual is primarily concerned with the mental states he is conscious of. Borges's playwright, for instance, wants to compose his work without anyone knowing about it; Thoreau wants to maintain his view that the state has no right to demand tax and wants to transmit his ideas to other people. Now, today's devices and new neural prostheses are able to detect brain data in the form of connection patterns and activation of nerve cells (cf. Peña-Gómez et al., 2017). Such patterns are probably the neuronal correlates of the thoughts (mental states) that the individual has at any point in time. The relationship between neural activation patterns and thoughts is being actively researched and, even though many aspects are still to be clarified, research is making a lot of advancements. It has been recently made it possible to decode what the brain is neurally representing by using artificial intelligence to interpret fMRI scans of subjects watching videos. Researchers used convolutional neural networks, a form of deep-learning (Wen et al., 2017). Their findings make it necessary to consider the ethical implications of this kind of “mind-reading”: such a device should be used for shared purposes and not to threaten individual privacy and freedom.

However, even brain data that do not match individual states and mental contents may be relevant to the individual, who may very well want to avoid both their diffusion and alteration (Mitchell et al., 2008). These elements, in fact, make it possible to predict behavior based not so much on conscious desires and intentions, but on “automatic” behavioral reaction patterns, such as what we call impulsivity or poor empathy. In addition, other techniques make it possible to identify the individual's unconscious tendencies manifested in the form of fast brain activation in front of a stimulus (such as for example, the so-called unconscious racism or other judgments we suppress in favor of responses that are more appropriate to the given social context). All this intuitively constitutes the individual's “private repository” of his convictions and identity, his dignity, and autonomy. Without it, the person may end up in a state of complete subjection to other individuals.

For sake of completeness, it should be specified that, from a philosophical standpoint, there is another aspect of mental life that, by definition, is inaccessible to external decoding, namely that of first-person sensations: what philosophy of mind calls “what it is like.” I am referring to things like perceiving a certain color nuance, enjoying a given wine, but also feeling loved or finding the solution to a mathematical problem (Nagel, 1974). Yet, from a scientific standpoint, the fact that one cannot feel the same first-person pleasure as another does not imply that we cannot decodify it. In fact, knowledge of the link between a stimulus (e.g., drinking a certain wine) and the activation of specific brain areas can make us validly infer that the subject is having, say, a reaction of disgust, even if she tries to hide it. In this sense, although we cannot access the subject's first person phenomenology, we can still decode her subjective cerebral/mental states and this makes the decoding of brain states relevant to the present discussion. That being said, there are many examples of situations where mental/brain integrity is at risk. The term “mental integrity” seems to be the most suitable way to account for the concept I want to express here. In the normative sense, the following definition might be adopted:

Def1: Mental Integrity is the individual's mastery of his mental states and his brain data so that, without his consent, no one can read, spread, or alter such states and data in order to condition the individual in any way.

This definition broadens the one offered by Ienca and Andorno (2017), by which the right to mental integrity “should provide a specific normative protection from potential neurotechnology-enabled interventions involving the unauthorized alteration of a person's neural computation and potentially resulting in direct harm to the victim.” The definition I propose addresses both the issue of privacy and that of cognitive freedom, which Bublitz defined as “the right to alter one's mental states with the help of neurotools as well as to refuse to do so” (Bublitz, 2013). My point is that privacy, understood as the secrecy of one's brain data and mental contents, is key to a free conduct, because autonomy is exercised not only in public but also in private. Being spied on through mind-reading reduces the subject's autonomy in the Kantian sense, that is, the subject can be limited in self-imposing her own norm of conduct, free of external pressures and conditioning (which are only avoided by keeping one's thoughts private). What follows is an inappropriate imposition over the individual, even in the absence of direct harm.

The definition I propose also grasps another aspect: as previously noted, even brain data apparently unrelated to conscious contents or cognitive processes (mental states) can help predict one's behavior. This is of particular importance in the light of the fact that recent interpretations of brain activity are emphasizing its predictive character. In particular, it has been argued that our brains are similar to prediction machines, capable of anticipating the incoming streams of sensory stimulation before they arrive (Hohwy, 2013; Clark, 2016). If the brain is more than a response machine and if actions are more than stimulus responses, but are a way of selecting the next input, then knowing the present state of the brain (understood as prediction machine) can say a lot about an individual and her future behavior—much more than one would have thought within a different paradigm of brain functioning. However, one may wonder why mental (cerebral) integrity should be granted special value compared to other individual aspects, and whether the right to integrity is relative or absolute. I will reply to the first question in this section, while the second will be tackled in the following one.

Mental integrity is the basis for freedom of thought as it was classically conceived, before the era of neuro-technological pervasiveness (Shen, 2013). It is the first and fundamental freedom that the individual must be granted in order to have all the other freedoms considered relevant. In the long Western tradition from Socrates to Augustine of Hippo, the inner life—the one that no one can see and with which no one can interfere—has always been considered the most precious and intangible resource of the human being. Personal autonomy seems to directly follow from freedom of thought. Human flourishing in many of its declinations—albeit not all of those proposed in different human cultures—feeds on freedom of thought understood as a “private repository” where nothing and no one can go without the person's consent. In fact, it has been claimed that “the right and freedom to control one's own consciousness and electrochemical thought processes is the necessary substrate for just about every other freedom” (Sententia, 2004). In this sense, cognitive freedom is a new form of freedom of thought that “takes into account the power we now have, and increasingly we will have, to monitor and manipulate cognitive function” (Ib.).

Faced with the potential new threats to mental integrity due to neuroscientific techniques, attempts have been made to introduce new human rights, specifically aimed at safeguarding the right to cognitive freedom, the right to mental privacy, the right to mental integrity, and the right to psychological continuity (Ienca and Andorno, 2017). As said, it is also important to protect brain data that can give access to the (more or less precise) prediction of the person's behavioral patterns through her neural connection and activation patterns. In fact, such information can be used for forms of prevention/discrimination (cf. Raine, 2013) based on the subject's hypothetical attitudes, or even to implement forms of forced re-education or compulsory moral bio-enhancement (cf. Persson and Savulescu, 2012; Douglas, 2014; cf. also Bublitz and Merkel, 2014).

In this vein, examples of mental integrity violations are related to brain-hacking. Ienca and Haselager (2016) define brain-hacking as “illicit access to and manipulation of neural information and computation.” Given that neural computation underlies cognition, behavior and our self-determination, any unauthorized access, as said, may have significant consequences. Think, for example, of people who have public responsibilities and who have to resort to devices to stabilize or (in the future) improve their brain functionality. They would certainly have to disclose the use of such prostheses (Garasic and Lavazza, 2016), but access to incoming and outgoing data could be a very serious violation of mental integrity for the implanted subjects. Indeed, one could use these data to infer—arbitrarily and without scientific certainty—tendencies or behavioral predispositions of the subject, both innate and due to incipient pathological states. And the disclosure of these news could seriously harm the subject with regard to his or her public role.

## The technical principle of protection of mental integrity

If the premise on the importance of mental integrity obtains, a (technical) principle for the protection of mental integrity may follow.

Def2: The technical principle for the protection of mental integrity is a functional limitation that should be incorporated into any devices capable of interfering with mental integrity (as defined in Def1). Specifically, new neural prostheses should (a) incorporate systems that can find and signal the unauthorized detection, alteration, and diffusion of brain data; (b) be able to stop any unauthorized detection, alteration, and diffusion of brain data. This should not only concern individual devices, but act as a general (technical) operating principle shared by all interconnected systems that deal with decoding brain activity.

As for devices already in use, they could, for example, incorporate a device emitting a signal with a certain wavelength through a mind-reading device detector that would be readily available to the public. This should respond to the need to prevent people from being secretly subjected to or deceived about the use of devices capable of threatening the right to mental/brain integrity (Ienca and Haselager, 2016). For example, think of neural prostheses for cognitive enhancement, especially for those who in the future might be obliged to use them because of their profession (airplane pilots, surgeons, etc.) and might want to avoid privacy violation or brain manipulation (Santoni de Sio et al., 2014). In addition, brain-altering devices could be equipped with various usage thresholds, each activated only with different access keys, related to the professional profiles of users: laboratory technicians, doctors, medical investigators… In this way, the most invasive practices would be only available to a few people that would have been selected over time for their professional skills and ethical integrity, as a safeguard against possible abuse.

As for new neural devices and prostheses (Lebedev et al., 2011)—such as tools for the treatment of consciousness disorders, direct brain-to-brain communication, networks composed of many brains, artificial parts of the brain replacing damaged circuitry and improving the existing one, and even the transfer of brain content and functions to an artificial carrier—it should be made possible to extract brain data only by means of special access keys managed exclusively by the subjects under treatment or by their legal representatives. Some of these devices could be specifically aimed at brain alteration for rehabilitation or other medical purposes, and likewise they should only be made accessible to professionals in charge of their correct use, which should be able to be monitored by a specific authority upon the subject's request. Think, for example, of new portable devices such as fNIRS instrument for mobile NIRS-based neuroimaging, neuroergonomics, and BCI/BMI applications (von Lühmann et al., 2015).

As for the military or anti-terrorism use of such devices, it would be desirable to stipulate an international treaty on the model of those that have been signed by many countries concerning antipersonnel mines, cluster bombs, or even chemical weapons. These treaties establish the dismantling and non-production of such weapons in peacetime. In fact, if such weapons were readily available during a war, countries would likely be tempted to use them. If the production of those weapons, however, had been interrupted long before, it would be much more difficult to resort to them. Similarly, if mind-reading or brain-altering devices were available without mechanisms preventing their improper use, some would likely want to use them for military purposes. This would be very tempting, because the point would not be to threaten anyone's safety, but only to temporarily violate their mental privacy and/or integrity in order to defend the community—say, from a terror attack. However, if unregulated mind-reading devices (that is, without the limits imposed by the technical principle proposed in Def2) were not available, it would be much more difficult to resort to them.

Notwithstanding all this, in some medical, legal, and military cases the right to mental integrity may seem to be competing with other rights, so that the principle of protection of mental integrity may be occasionally bypassed. Therefore, it must be established whether the right to mental integrity is absolute or relative. To assert that the right to mental integrity is absolute requires a strong justification covering every possible case—a goal that is hard to reach and is unlikely to have many supporters. On the other hand, setting the right to mental integrity as relative risks weakening it, making it less important than it is and making it much easier to violate thanks to neurotechnological progress. Consider the positive purposes for which neural prostheses were built in the first place: they might be partly hindered by the technical principle of the protection of mental integrity. For example, prostheses able to signal and/or prevent epileptic crises might also be able to do the same for violent outbursts. But these positive goals should only be pursued if the subject in question has expressed full informed consent on the matter.

Even in this case, however, it might be argued that the individual's freedom, autonomy, and intrinsic value are only expressed when the subject enacts positive behaviors himself, without being constrained by an automated device. In other words, if a person suffering from epilepsy may give her informed consent to treatment, being fully aware of its pros and cons, the issue seems a lot more complex when it comes to a violent subject. For example, he may find himself forced to choose between staying in prison or accepting a neural prosthesis controlling his violent drives; but it must be considered that violence may sometimes be needed to defend oneself or others from a threat, so that having the drive controlled at a neural level might be highly dysfunctional and damaging to the individual himself.

Other cases may involve different levels of consensus and collective security. For example, access to the EEG data of a vehicle driver could allow for a built-in tool to detect a neuronal activation pattern that would lead to decreased attention while driving (Biondi and Skrypchuk, 2017). The purpose of avoiding serious accidents could be considered superior to the driver's right not to undergo the constant monitoring of his brain states. Following the same line of thought, protecting the population from terror attacks could result in introducing compulsory “neural” control in order to find potential terrorists. In this case, the right to mental integrity would be violated for a number of people, most of whom would be unrelated to any malicious plans. Generally speaking, it might be argued that the best solution would be to let judges decide on a case by case basis whether to authorize the violation of the right to integrity.

Finally, one may wonder how the need to protect the fundamental right to mental integrity may coexist with the need to violate the following technical principle. Well, in this case, the increasingly sophisticated neurotechnological techniques might help. In fact, producing all neural prostheses from the start according to the technical principle for the protection of mental integrity would make it more complicated to use them in violation of this fundamental right, even if for a goal that is considered socially positive. For example, if the secret codes to control neural prostheses were available to an independent authority, this would make potential breaches more rare. Also, the process of authorization of “improper” but “necessary” use of such prostheses—in cases related to collective security—would be more difficult and rigorous. One can also support a more rigid position, so as to further limit the possibility of mental integrity violations. For example, one can argue that, once implanted, these devices should belong to, and only be “controlled” by, the subject, even in case of maintenance and reprogramming. However, the subject will always need physicians and experts to implant the prosthesis, make it work and introduce potential changes. The problem therefore does not seem to have an easy solution.

## The case of closed-loop DBS

A new type of deep brain stimulation (DBS) may be a good illustration of the issues addressed so far as well as of other ethical quandaries associated with them. DBS, consisting of an external neurostimulator that sends electrical impulses to brain nuclei (according to the pathology to be treated) through electrodes implanted permanently, is used to reduce the symptoms of Parkinson's disease, dystonia, chronic pain, major depression, and obsessive–compulsive disorder. The results, especially for the treatment of neuropsychiatric conditions, such as Tourette's syndrome, are mixed (Greenberg et al., 2010; Dougherty et al., 2015). The first generation of BDS is called “open loop,” as the devices provide a given level of brain stimulation, which can be modified over time to try to improve the therapeutic effect, but feedback is linked to the patient's subjective assessment. Also, in the presence of side effects, the stimulator can be turned off, thereby making the DBS a reversible stimulation technique.

The new generation of DBS devices is instead called “closed-loop,” as electrodes can stimulate the areas in which they are placed and record their electrical activity. The idea is that sensors can detect brain activity patterns that are related to the symptoms to be cured, using these data at a neural level to adjust the stimulation accordingly. This makes feedback much faster: indeed, it should hopefully be simultaneous to, if not preventive of, the emergence or deterioration of the neuropsychiatric symptoms to be treated. In addition, the patient and physicians allegedly don't have to do anything, as happens with diabetic patients who use automated insulin pumps (Wheeler et al., 2015).

The new tools are inspired by brain-computer interfacing, where algorithms are used to decode brain activity and identify impaired patients' intentions to control prostheses or other devices (Widge et al., 2014). Once the electrical activity is registered, the aim is to classify it as either a “healthy” or a “symptomatic” state, and to modify the location and intensity of the stimulation accordingly. The first results were achieved with epilepsy and tremor control (Malekmohammadi et al., 2016).

These devices or prostheses raise new ethical questions (Kellmeyer et al., 2016; Goering et al., 2017) and several orders of issues. The first order of questions concerns the personal condition of the implanted subject, with respect to her identity, agency, and autonomy. The second, closely linked to the former, concerns the protection of mental integrity and freedom of thought. The third concerns the liability for violations and malfunctions that may have consequences on the subject and on third parties.

Let's start with the first issues: namely, the identity, agency, and autonomy of the implanted subject. First-generation DBS already triggered a wide debate on the possible modification of the subject's identity. For example, Schechtman (2009) has made interesting remarks on the deep brain stimulation used for Parkinson's. Neurosurgery can cause changes in the patient's character and inclinations. Schechtman believes that DBS can threaten personal identity because it can change—partly or fully—the subject's personality traits, aims, and interests. According to her, if, after undergoing DBS, a patient shows a very different behavior, it can be said that he is, in a way, “a different person”. What matters is *how* the patient has changed: not as a result of what he has seen, learned or thought about, but through the direct effect of a passively undergone deep brain stimulation.

Schechtman sets two constraints to be respected in the light of narrative identity. The first is the “reality constraint,” according to which the narrative of the self-making up a person's identity should be consistent with external reality. The second is the “articulation constraint,” according to which the self-narrative should be constructed by the subject in a way that justifies his choices and behaviors. Schechtman (2009) claims that “the mechanism of personality change is important to its effect on forensic personhood and identity.” In fact, if a patient treated with DBS were to show a personality change, one would “have to acknowledge that his current passions and interests—the things he takes as reasons—were caused by the manipulation of his brain.” As a consequence, this change would have to be considered “disruptive to his forensic personhood and identity in a way that natural personal development would not have been” (Schechtman, 2009).

However, it has been claimed (Müller et al., 2017) that Schechtman's objection is a case of naturalistic fallacy, entailing confusion between the property of being natural and that of being good. Indeed, before being subjected to the BDS implant a person may judge that improving his pathological condition is worth the trade off with some of his personality aspects. Subsequently, however, the person may not be able to assess their condition, although they may be helped by relatives and friends to understand and evaluate changes in their identity (impulsivity, hypersexuality, gambling; cf Glannon, 2009). The matter seems particularly complicated, and closed-loop DBS may raise further questions in this regard. Indeed, there is a degree of automaticity in the stimulation adjustments (resulting in a possible accentuation of the change of personal identity) which makes the implant even more difficult to endorse by the implanted subject.

However, precisely because personal identity is something that is difficult to define with precision and is subject to continual adjustments, also due to external situations outside of the subject's control, many scholars believe that the threats to the agency and the autonomy of the subject are more worrying than the ones to personal identity. For example, Baylis (2013) believes that DBS is problematic “insofar as it is a threat to agency—the ability to make informed and rational choices—as when a person's actions do not flow from her intentions or beliefs but rather are the result of direct brain manipulation.” In this sense, closed-loop BDS can be a special threat to the subject's agency and autonomy, “generally understood to refer to the capacity to be one's own person, to live one's life according to reasons and motives that are taken as one's own and not as the product of manipulative or distorting external forces” (Christman, 2015, p. 1). Consider an extension of a real case reported by Klein et al. (2016). A person treated for depression with closed loop DBS could go to the funeral of a dear friend and realize that she is not really sad for the loss and, indeed, that she has said and done something socially and interpersonally inappropriate. Since the device “reads” the aggravation of depression, it intervenes to counteract it, in this case “against the will” of the subject, who appears to other people as detached or insensitive, or in any case not in tune with the circumstance.

As for violations of mental integrity and freedom of thought, the closed loop BDS's ability to record brain activity and act in real time consequently leaves room for unauthorized and malicious actions that can strongly affect the subject's identity, agency, and autonomy. For this new generation of DBS, it seems even more advisable to apply the functional technical principle that aims to (a) incorporate systems that can find and signal the unauthorized detection, alteration and diffusion of brain data and brain functioning; (b) and provide means, within the same device or connected to it, able to stop any unauthorized detection, alteration, and diffusion of brain data. Specifically, implanted subjects would find it difficult to realize that their device had been hacked, as it would respond to changes in neuronal activity that are only instrumentally detectable. In this way, behavioral changes that were driven by malicious subjects able to take control of the device could not be easily discovered, neither by the implanted subject nor by the people around him. In fact, brain-hacking-induced behavioral effects would hardly be distinguished from those induced by stimulation as a side effect of the cure.

This leads to the issue of liability for potential malfunctions or misuse of devices. It is known that drug companies have been prosecuted by family members of Parkinson's patients who, under the influence of dopamine agonists, have squandered their wealth with gambling. If closed-loop DBS caused malicious or dysfunctional behaviors that were not foreseen or reported as potential side effects, the manufacturer could be considered responsible, in part or in full. However, it presently seems difficult to evaluate how a subject may react to stimulation, considering the extreme variability reported in literature (Morishita et al., 2014). Additionally, responsibility could extend to physicians who prescribed and implanted the device, as they were also supposed to evaluate its appropriateness and potential consequences.

All this supports the idea that regulatory bodies, specifically those who are called upon to endorse the trade of clinical devices, should implement strict rules for both the subject's safety and the protection of his mental integrity and freedom of thought. However, the extreme importance of the issues covered in this paper could also suggest the idea of special agencies (including supranational ones) with the task of supervising the development and marketing of neural prostheses. This should not be understood as a limitation to research and to the efforts to apply new knowledge for the treatment of an increasing number of pathologies. Rather, it should be seen as a means to protect the patients, so that their mental integrity and freedom of thought are kept safe.

Also, one could consider the distinction between neural prostheses used for clinical purposes and neural prostheses used by healthy subjects for the purpose of sports or cognitive enhancement. Devices that are less invasive than DBS are already being tested, and closed-loop BMI systems have already been experimented in healthy and clinical populations, especially the real-time EEG-Based Brain Machine Interface (BMI) used for gait rehabilitation in individuals who have problems with bipedal locomotion (e.g., Luu et al., 2016, 2017). In addition, researchers could construct devices able to capture a decrease in attention thanks to neuronal activation patterns detected through EEG, and to act immediately, without the conscious intervention of the subject, with a stimulation via tDCS. Such tools would need the same regulations as medical devices, but given that the subjects in question would not have specific medical issues, perhaps this regulation could be less strict. Still, malfunctioning and misuse should still be prosecuted and sanctioned. Criminal law provisions would ultimately be the main deterrent and punitive tool for the use of neural prostheses to enhance physical and cognitive performances and other recreational uses. However, it should not be excluded that the spread of these not clinical uses might be followed by a stricter protection of users.

Finally, and surprisingly, a new field in which freedom of thought and mental privacy and integrity could be endangered is that of neuroaesthetics and creativity. There has been growing interest in understanding the neural basis of aesthetic perceptions and creative abilities both of lay people and professional artists. The risk does not come from the collection of data as such, but from the use that could be made of it. New knowledge is the result of studies that are conducted not only in the laboratory, but also in “natural” environments such as museums.

For example, Kontson et al. (2015) collected aesthetic and emotional preference data from over 400 museum visitors using portable EEG systems. The researchers were able “to quantify the aesthetic experience through the analysis of brain differences that arise between participants during è an aesthetic judgment, and through functional network connectivity” (Kontson et al., 2015). It was thus possible to find quantitative differences in EEG signals across subjects; patterns of brain activity associated with emotionally stimulating and aesthetically pleasing pieces, and putative networks engaged in the perception and judgments of art stimuli. Vessel et al. (2012), combining fMRI and behavioral analysis of individual differences in aesthetic response, identified “two distinct patterns of neural activity exhibited by different sub-networks. Activity increased linearly with observers' ratings (4-level scale) in sensory (occipito-temporal) regions (…). In contrast, a network of frontal regions showed a step-like increase only for the most moving artworks (“4” ratings) and non-differential activity for all others.”

Based on the EEG data, one might create a neural map, showing what regions of the brain are elicited to activate by different features of a specific aesthetic experience. The exact reconstruction of the cerebral maps of aesthetic perception could allow the exploitation of this knowledge to induce aesthetic appreciation in the visitor or in the consumer, combining salient elements that do not “objectively” constitute a work of art but that still arouse specific brain activations. This would produce unwanted external control over aesthetics or creative thought, which certainly would affect people's freedom of thought and therefore mental integrity. Just as the diffusion of certain perfumes can induce emotional states that trigger certain behaviors to the detriment of others, so the presentation of visual or acoustic elements that can activate specific cerebral networks might orient people's aesthetic judgment unbeknownst to them. On the other hand, there will be positive effects if individuals become able to consciously increase their creativity on the basis of the knowledge of the brain mechanisms that allow us to appreciate and (positively or negatively) judge an artifact or intellectual work such as a novel or a symphony.

## Conclusion

In this paper—which has only envisioned potential problems and solutions to be further investigated—I have tried to show that there is a fundamental right to mental integrity that may be threatened by the development of increasingly sophisticated neural prostheses, able to detect and alter neural connection and activation patterns. I have therefore argued for the introduction of a technical principle to protect the right to mental integrity, which would undermine any attempt to violate such fundamental right.

However, the right to mental integrity may not be seen as absolute if other equally important goods or values (like physical safety) are at high risk. In such situations, the proposed general functional principle may still be useful in limiting the extent of the violation of the right to integrity in favor of other rights, goods, or values. The research on and development of neural prostheses should therefore consider the implications of potential violations on mental integrity, trying to incorporate the functional technical principle to protect it. Even if not fully implemented, this principle can still be a reference point for the public discussion of the ethical, legal, and social consequences of the introduction of new invasive neural prostheses.

## Author contributions

The author confirms being the sole contributor of this work and approved it for publication.

### Conflict of interest statement

The author declares that the research was conducted in the absence of any commercial or financial relationships that could be construed as a potential conflict of interest.
